# Case report: A novel variant (H49N) in *Myelin Protein Zero* gene is responsible for a patient with Charcot–Marie–Tooth disease

**DOI:** 10.3389/fneur.2024.1319962

**Published:** 2024-02-28

**Authors:** Gao-Hui Cao, Mei-Fang Zhao, Yi Dong, Liang-Liang Fan, Yi-Hui Liu, Yao Deng, Lu-Lu Tang

**Affiliations:** ^1^Department of Cell Biology, School of Life Sciences, Central South University, Changsha, China; ^2^Department of Neurology, Affiliated Hospital of Yangzhou University, Yangzhou, China; ^3^Department of Cardiovascular Surgery, National Clinical Research Center for Geriatric Disorders, Xiangya Hospital, Central South University, Changsha, China

**Keywords:** *Myelin Protein Zero*, Charcot–Marie–Tooth disease, CMT dominant intermediate D, missense variant, whole exome sequencing

## Abstract

This report presents a case of Charcot–Marie–Tooth dominant intermediate D (CMTDID), a rare subtype of Charcot–Marie–Tooth disease, in a 52 years-old male patient. The patient exhibited mobility impairment, foot abnormalities (pes cavus), and calf muscle atrophy. Whole exome sequencing and Sanger sequencing suggested that a novel variant (NM_000530.8, c.145C>A/p.His49Asn) of *MPZ* may be the genetic lesion in the patient. The bioinformatic program predicted that the new variant (p.His49Asn), located at an evolutionarily conserved site of *MPZ*, was neutral. Our study expands the variant spectrum of *MPZ* and the number of identified CMTDID patients, contributing to a better understanding of the relationship between *MPZ* and CMTDID.

## 1 Introduction

Charcot–Marie–Tooth disease (CMTD) encompasses a genetically heterogeneous group of disorders called hereditary sensory and motor neuropathies that damage the peripheral nerves (1, 2). The typical symptoms of CMTD include muscle atrophy in the feet, pes cavus, and decreased sensitivity to touch, heat, and cold in the feet and lower legs (3). Other symptoms, including hearing loss, scoliosis, hip dysplasia, restless legs syndrome, and tremor, can also be present in CMTD patients (3). As the most common inherited disorder involving the peripheral nerves, the prevalence of CMTD is about 1 in 2,500 worldwide (4). Currently, variants in four genes (*Peripheral Myelin Protein 22, Gap Junction Beta 1, Myelin Protein Zero, and Mitofusin 2*), are responsible for over 90% of CMT patients (5).

The *Myelin Protein Zero* (*MPZ*) gene is located on chromosome 1q23.3, and it consists of 6 exons, spanning approximately 6,369 kilobases. This gene is specifically expressed in Schwann cells of the peripheral nervous system and encodes a type I transmembrane glycoprotein that is a major structural component of the peripheral myelin sheath (6, 7). Acting as an adhesion molecule, the MPZ protein functions like molecular glue, playing a role in tightly packing the myelin around nerve cells, which wrap around and insulate peripheral nerves (7). Currently, approximately 5% of CMTD patients result from variants in *MPZ* (8–10). Additionally, some studies have also reported that *MPZ* variants can lead to other polyneuropathies, such as Dejerine–Sottas syndrome and congenital hypomyelinating nesuropathy (11, 12).

Here, we studied a Chinese family presenting with distal atrophy and weakness. Whole exome sequencing revealed a novel variant (NM_000530.8, c.145C>A/p.His49Asn) in the *MPZ* gene within the proband. Sanger sequencing additionally confirmed the presence of this novel variant in other affected family members, suggesting co-segregation. Furthermore, bioinformatics software predicted that this newly identified *MPZ* variant is deleterious.

## 2 Case report

### 2.1 Clinical description

**Figure 1:**
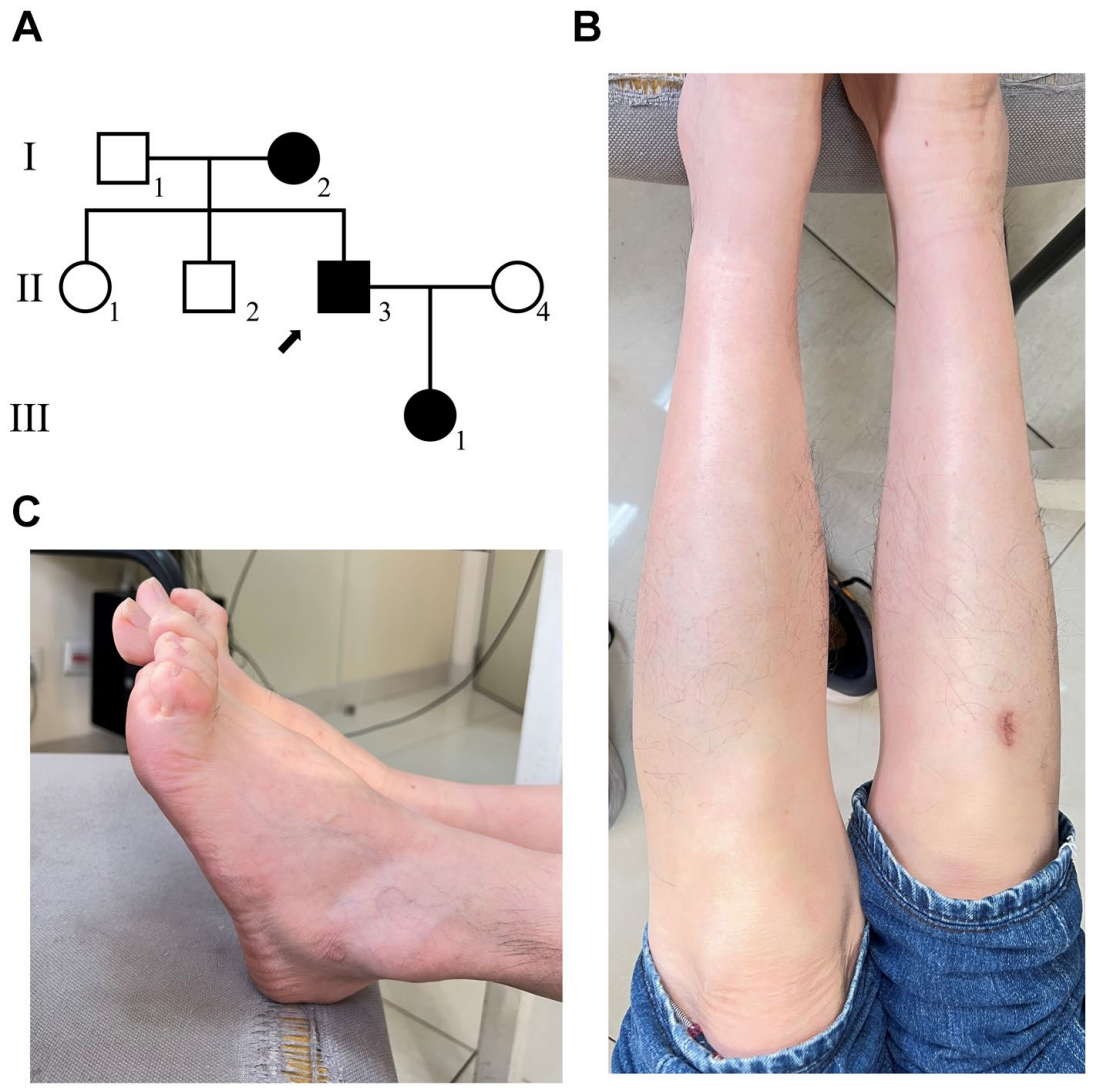
The clinical diagnosis of the proband. **(A)** Family diagram of patients with disease, and the arrow shows the proband. **(B)** The morphology of the patient’s feet has significantly high arches. **(C)** The patient has calf muscle atrophy.

The family, including seven people were investigated in this study (Figure 1A). The proband (II-3), a 52 years-old male from Jiangsu province in eastern China. The proband came to our clinic 1 year ago (November 2022). According to his own account, he began to realize that exercise was more difficult seven years ago (at the age of 45). The condition slowly worsened until it was difficult to walk, so he came to our hospital for consultation.

Clinical examination reveals: the proband’s lower limbs exhibit an inverted bottle shape, noticeable atrophy of the calf muscles, and spinal curvature. The proband has no dislocation, but the arches are markedly elevated, displaying claw-like deformities in the toes. The patient experiences difficulties in movement, demonstrating an abnormal striding gait and poor limb balance. The patient has weaknesses in the lower limb muscles, reduced strength in the arms, but no limb tremors. Both Achilles tendon reflex and knee-jerk reflex are diminished, and there is a decrease in pinprick sensation (Figures 1B,C). Electromyography (EMG) indicated multiple symmetrical peripheral nerve lesions, particularly myelin damage. Peripheral motor nerve conduction velocity (MNCV) was moderately impaired (Table 1), alongside reduced sensory nerve conduction velocities (SNCV) in the upper limb (Table 2). In addition, autoimmune peripheral neuropathy and paraneoplastic nerve syndrome were excluded by examining ganglioside antibody spectrum (GM1, GD1b, GQ1b, GM2, GM3, GD1a, GT1b, Sulfatide, GM4, GD2, GD3, GT1a) and paraneoplastic nerve syndrome spectrum [Hu, Yo, Ri, CV2, PNMA2 (Ma-2/Ta), amphiphysin, recoverin, SOX1, titin, Zic4, GAD65, Tr (DNER)] in serum and cerebrospinal fluid. A family history investigation indicated that his mother (I-2) also suffered from mobility impairment and calf muscle atrophy. Additionally, his daughter (III-1) occasionally experiences muscle weakness in her limbs.

**Table 1:** Motor nerve conduction velocities of EMG result.

MNCV		Latency (ms)	Amplitude (mv)	CV (m/s)	F-wave latency (ms)
Ulnar nerve. Left	Wrist-ADM	2.99	7.80		40.30
	Below elbow-wrist	11.20	5.90	34.10	
Ulnar nerve. Right	Wrist-ADM	3.85	5.60		42.40
	Below elbow-wrist	11.30	5.10	37.60	
Median nerve. Left	Wrist-APB	6.57	3.50		44.50
	Elbow-wrist	13.10	2.50	38.30	
Median nerve. Right	Wrist-APB	5.45	5.10		45.10
	Elbow-wrist	12.80	4.10	34.00	
Tibial nerve. Left	Ankle-AH	5.58	9.30		52.00
	Popliteal-ankle	18.90	5.30	34.50	
Tibial nerve. Right	Ankle-AH	6.35	6.90		52.70
	Popliteal-ankle	21.50	3.40	27.10	
Nervi peroneal is common. Left	Below knee-tibialis anterior	4.52	2.30		
	Upon knee-below knee	6.90	2.20	42.00	
Nervi peroneal is common. Right	Below knee-tibialis anterior	6.96	0.75		
	Upon knee-below knee	9.76	1.39	35.70	

**Table 2:** Sensory nerve conduction velocities of EMG result.

SNCV		Latency (ms)	Amplitude (mv)	CV (m/s)
Ulnar nerve. Left	Finger V-wrist	4.79	2.1	24.7
Ulnar nerve. Right	Finger V-wrist	4.25	3.8	34.6

As a result, patients are diagnosed with Charcot–Marie–Tooth disease. CMT cannot be effectively treated, so none of the patients were hospitalized. Patients are advised to increase their intake of vitamins B1 and B12. A follow-up was conducted 1 year later. There was no significant change in the patient’s condition.

### 2.2 Genetic analysis

**Figure 2:**
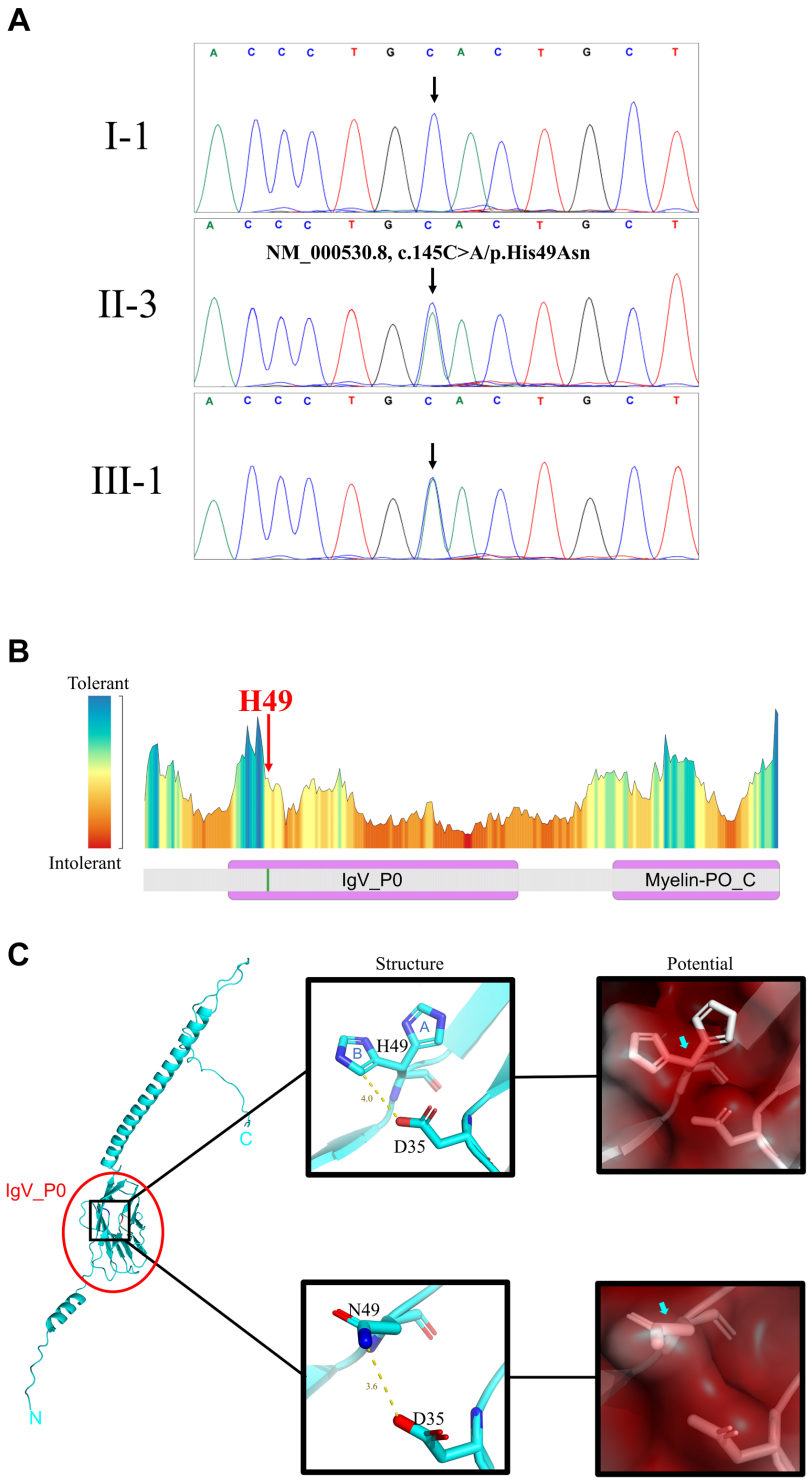
Gene and protein analysis. **(A)** Sanger sequencing results for I-1 (unaffected +/+), II-3 (affected +/−), and III-1 (affected +/−) patients (see Figure 1A for pedigree). **(B)** Predicted residue tolerance in the MPZ protein obtained using MetaDome. H49 is predicted to be of neutral tolerance to mutations. MPZ contains two domains: immunoglobulin-like domain of protein zero (IgV_P0) and Myelin-PO glycoprotein cytoplasmic C-term (Myelin-PO_C) by Conserved Domain Search prediction. **(C)** H49 is located on the IgV_P0 domain shown on the full length MPZ model (left panel). The atomic conformation (middle panels) and the surface electrostatic potential energy (right panels) change with the H49N variant. In the middle panels, blue “A” and “B” indicate 2 conformations of H49 in the crystal structure. Yellow line and text indicate the distance between atoms on side chains at positions 35 and 49 (angstrom). The blue and red atoms represent negative and positive charges, respectively. The variation of H49N causes the side chain charge from negative to become neutral. In the right panels, red means positive, blue means negative, and white means neutral. The surface potential prediction is slight changed from positive to neutral.

Initially, multiplex ligation-dependent probe amplification was employed to exclude copy number variants in two candidate genes, *Kinesin Family Member 1B* and *Peripheral Myelin Protein 22*, which are commonly associated with copy number variations in CMTD patients (Supplementary Figure S1). Subsequently, the proband underwent whole sequencing to detect potential gene variants. A total of 9.16 GB of data, encompassing 70,012 SNVs/Indels, were identified in the proband. Following the aforementioned data filtering process, 12 variants were retained (Supplementary Table S1). Among these 12 variants, only the novel variant (NM_000530.8, c.145C>A/p.His49Asn) in *MPZ* was deemed to be the underlying genetic anomaly for the family. Sanger sequencing further confirmed the co-segregation of this variant with the affected family members (Figure 2A) and its absence in our 200 control cohorts. This novel variant, resulting in the substitution of histidine with asparagine, was located at a neutral tolerant site and Immunoglobulin-like domain of protein zero (IgV_P0) (Figure 2B). We predicted and compared the protein structure after the p.His49Asn variant (Figure 2C), based on the latest reports of IgV_P0 domain (PDBid: 8iia (13)). Surface potential analysis additionally revealed that variant altered the surface charge of the MPZ protein (Figure 2C). According to ACMG guideline, the variant belongs to Likely pathogenic (PM1 + PM2 + PP1 + PP3) (14).

## 3 Discussion and conclusion

Charcot–Marie–Tooth disease (CMTD) comprises several subtypes, including CMT dominant intermediate D (CMTDID), a rare form defined by motor nerve conduction velocity (MNCV) falling within the intermediate range of 25–45 m/s (1, 15). This subtype was initially reported in 1999 within a 4-generation Macedonian family. The family exhibited a symmetrical pattern of distal muscle atrophy, weakness, and sensory impairment, more pronounced in the lower limbs and to a lesser extent in the upper limbs, besides, the youngest patients only 34 years old (16). In our study, the proband showed myelin damage in both motor and sensory nerves, with MNCV ranging from 27–42 m/s. Whole exome sequencing and Sanger sequencing further confirmed that the *MPZ* variant (NM_000530.8, c.145C>A/p.His49Asn) was the genetic anomaly responsible for the family’s condition. Our research may broaden the variant spectrum of *MPZ* and aid in genetic counseling and early diagnosis for CMT disease patients.

MPZ protein, an integral membrane glycoprotein, primarily connects adjacent lamellae to stabilize myelin assembly (17). It serves as the principal structural component of peripheral myelin and is exclusively expressed in Schwann cells (18). The MPZ protein is composed of three domains: a singular Immunoglobulin V-Type-like extracellular domain, a lone transmembrane domain, and a single cytosolic domain (19). Previous studies have indicated that the majority of pathogenic *MPZ* variants can trigger the unfolded protein response and endoplasmic reticulum retention (7). In our investigation, the novel variant (NM_000530.8, c.145C>A/p.His49Asn) in *MPZ* was situated in the extracellular IgV_P0 domain (Figure 2B). Crystallographic analysis of the extracellular domain of MPZ revealed its capacity to form interactions, resulting in homotetramer structures which are supported by recent solution-based studies using SEC, SAXS, and NMR (13, 20). Further, one recent study indicated that the extracellular domains of the MPZ protein form an 8-mer responsible with a potential involvement in membrane adhesion (13). The novel variant’s alteration of the MPZ protein’s charge may potentially influence the stabilization of membrane layers in compact myelin and adhesion between layers, further leading to demyelination (Figure 2C). Also, MPZ plays a crucial role in the development of myelin structure. Variants in *MPZ* could potentially impact the normal formation of myelin, consequently disturbing the interactions between Schwann cells and axons, ultimately resulting in abnormal axon (7). Interestingly, the earliest identified CMTDID patients carried the D35Y variant (16). The shortest distance between D35 and H49 (B conformation) is 4 angstroms, and the shortest distance between D35 and N49 is only 3.6 angstroms (Figure 2C). Therefore, there may be a relationship between CMTDID disease and residue contact of these two positions. In addition, Veneri et al. (21) found that the increase of glycosylation sites in MPZ can impair its function and lead to loosen myelin. Mutations in H49N produce an NCS sequence that belongs to the glycosylation motif (N-X-S/T), resulting in excessively glycosylation of MPZ. CMTDID reported in this study belongs to the intermediate type (16), showing both mild demyelinating lesions and mild axonal abnormalities.

Currently, a total of 180 variants in the *MPZ* gene have been reported in patients displaying various phenotypes. Through summarizing these reported *MPZ* variants, we observed that the majority of cases (78.2%) carrying *MPZ* variants exhibited Charcot–Marie–Tooth (CMT) phenotypes. Additionally, 7.4% of carriers presented with Dejerine–Sottas syndrome, and 0.8% displayed Roussy–Levy syndrome. Within 78.2% of carriers manifesting CMT diseases, a mere 0.4% of patients showed the CMT dominant intermediate D (CMTDID) subtype (HGMD database: https://www.hgmd.cf.ac.uk/ac/index.php). This scarcity underscores the rarity of CMTDID subtypes identified among *MPZ* variant carriers. In this context, we identified a novel *MPZ* variant (NM_000530.8, c.145C>A/p.His49Asn) in a CMTDID patient, thereby reporting a unique case arising from a novel *MPZ* variant. This contributes to the expanding pool of recognized CMTDID patients and furthers our understanding of this subtype.

In MPZ+/− mice, neuropathy develops in adulthood, displaying minimal nerve conduction slowing and mild demyelination, akin to patients with *MPZ* variants (22, 23). Recently, Shackleford et al. created a new *MPZ* variant (p.T124M) knock-in mouse model, revealing impaired motor performance, reduced compound motor action potential amplitudes, and axonal damage, albeit with normal nerve conduction velocities (24). The distinctions between MPZ+/− mice and MPZ (p.T124M) knock-in mice underscore the intricate role of *MPZ* in CMT disease development, implying that this study’s primary constraint lies in its absence of functional research.

In summation, our study employed whole exome sequencing and Sanger sequencing to identify a novel *MPZ* variant (NM_000530.8, c.145C>A/p.His49Asn) in a Chinese family afflicted by CMT disease. Subsequent analysis validated this variant as the cause of a rare CMT subtype known as CMTDID. Our work enhances the diversity of *MPZ* variant profiles and the roster of recognized CMTDID patients, contributing to a deeper comprehension of the relationship between *MPZ* and CMTDID.

## Data availability statement

The datasets presented in this article are not readily available because of ethical and privacy restrictions. Requests to access the datasets should be directed to the corresponding authors.

## Ethics statement

The studies involving humans were approved by the Ethics Committee of the Affiliated Hospital of Yangzhou University, Yangzhou, China. The studies were conducted in accordance with the local legislation and institutional requirements. The participants provided their written informed consent to participate in this study. Written informed consent was obtained from the individual(s) for the publication of any potentially identifiable images or data included in this article.

## Author contributions

GH-C: Data curation, Formal analysis, Investigation, Software, Validation, Visualization, Writing – original draft. M-FZ: Data curation, Formal analysis, Investigation, Methodology, Software, Validation, Visualization, Writing – original draft. YDo: Writing – review & editing. L-LF: Methodology, Writing – review & editing. Y-HL: Writing – review & editing. YDe: Conceptualization, Funding acquisition, Project administration, Resources, Supervision, Validation, Writing – review & editing. L-LT: Conceptualization, Funding acquisition, Project administration, Resources, Supervision, Validation, Writing – review & editing.
