# Supplementary material for: Case report: A novel variant (H49N) in Myelin Protein Zero gene is responsible for a patient with Charcot–Marie–Tooth disease
Source: Front Neurol. 2024 Feb 28;15:1319962. doi: 10.3389/fneur.2024.1319962 (PMC10936578; doi:10.3389/fneur.2024.1319962)
Supplement: Supplementary file 1 [file Data_Sheet_1.zip › SupMethod.docx]

# Materials and Methods

## 1 Subjects

This study was approved by the Ethics Committee of the Affiliated Hospital of Yangzhou University, Yangzhou, China and performed in accordance with the principles enshrined in the Declaration of Helsinki. The participants provided their written informed consent to participate in this study.

The family including seven persons was investigated in this study (Figure 1A). The peripheral blood samples of three patients (I-2, II-3, III-1) and four healthy people (I-1, II-1, II-2, II-4) were collected and used to extract the Genomic DNA by DNeasy Blood & Tissue Kit (Qiagen 69504). Simultaneously, clinical data including the pictures and electromyography (EMG) were also collected.

## 2 Whole exome sequencing

The proband (II-3) was selected for whole exome sequencing. BerryGenomics Biotech company (Beijing, China) conducted exome capture, next-generation sequencing, and standard analysis, including variant annotation referring to Ensemble release 82, and filtering based on ANNOVAR[1] Documentation. The data filtering strategies employed were as follows: (a) Non-synonymous SNPs or frameshift-causing Insertion–deletion mutations (INDELs) with an alternative allele frequency > 0.05 in the NHLBI Exome Sequencing Project Exome Variant Server (ESP6500) (<https://esp.gs.washington.edu/drupal/>), dbSNP152[2], the 1000 Genomes project[3], the ExAC database[4], the gnomAD[5] database were excluded. (b) Filtered SNVs and INDELs predicted to be damaging by SIFT[6], PolyPhen-2[7], and Mutation Taster[8] were retained. (c) Co-segregation analysis was performed within the family through Sanger sequencing.

## 3 Mutation validation and bioinformatic analysis

All filtered mutations from the family members underwent validation through Sanger sequencing. Primer pairs were designed using Primer 5 (<https://www.bioprocessonline.com/doc/primer-premier-5-design-program-0001>). The sequences of the polymerase chain reaction (PCR) products were determined using the ABI 3100 Genetic Analyzer (ABI, Foster City, CA). (Figure 2A).

MetaDome[9] software (https://stuart.radboudumc.nl/metadome/) showed that the affected amino acids were in the region of neutral tolerance. Conservation analysis was conducted using the ConSurf Server[10] software. Domain Search has done by NCBI Conserved Domain Search[11]. (Figure 2B).

The Alphafold2 [12] and Swiss-Model[13] was employed to identify the mutation's impact on protein function. The APBS[14] plug-in was used to map the protein surface potential in PyMOL. (Figure 2C).

# Methods reference

[1] K. Wang, M. Li, and H. Hakonarson, ANNOVAR: functional annotation of genetic variants from high-throughput sequencing data. Nucleic Acids Res 38 (2010) e164.

[2] S.T. Sherry, M.H. Ward, M. Kholodov, J. Baker, L. Phan, E.M. Smigielski, and K. Sirotkin, dbSNP: the NCBI database of genetic variation. Nucleic Acids Res 29 (2001) 308-311.

[3] A. Auton, L.D. Brooks, R.M. Durbin, E.P. Garrison, H.M. Kang, J.O. Korbel, J.L. Marchini, S. McCarthy, G.A. McVean, and G.R. Abecasis, A global reference for human genetic variation. Nature 526 (2015) 68-74.

[4] ExAC project pins down rare gene variants. Nature 536 (2016) 249.

[5] L. Koch, Exploring human genomic diversity with gnomAD. Nat Rev Genet 21 (2020) 448.

[6] P.C. Ng, and S. Henikoff, SIFT: Predicting amino acid changes that affect protein function. Nucleic Acids Res 31 (2003) 3812-3814.

[7] I. Adzhubei, D.M. Jordan, and S.R. Sunyaev, Predicting functional effect of human missense mutations using PolyPhen-2. Curr Protoc Hum Genet Chapter 7 (2013) Unit7.20.

[8] R. Steinhaus, S. Proft, M. Schuelke, D.N. Cooper, J.M. Schwarz, and D. Seelow, MutationTaster2021. Nucleic Acids Res 49 (2021) W446-W451.

[9] L. Wiel, C. Baakman, D. Gilissen, J.A. Veltman, G. Vriend, and C. Gilissen, MetaDome: Pathogenicity analysis of genetic variants through aggregation of homologous human protein domains. Hum Mutat 40 (2019) 1030-1038.

[10] H. Ashkenazy, E. Erez, E. Martz, T. Pupko, and N. Ben-Tal, ConSurf 2010: calculating evolutionary conservation in sequence and structure of proteins and nucleic acids. Nucleic Acids Res 38 (2010) W529-W533.

[11] S. Lu, J. Wang, F. Chitsaz, M.K. Derbyshire, R.C. Geer, N.R. Gonzales, M. Gwadz, D.I. Hurwitz, G.H. Marchler, J.S. Song, N. Thanki, R.A. Yamashita, M. Yang, D. Zhang, C. Zheng, C.J. Lanczycki, and A. Marchler-Bauer, CDD/SPARCLE: the conserved domain database in 2020. Nucleic Acids Res 48 (2020) D265-D268.

[12] K. Tunyasuvunakool, J. Adler, Z. Wu, T. Green, M. Zielinski, A. Žídek, A. Bridgland, A. Cowie, C. Meyer, A. Laydon, S. Velankar, G.J. Kleywegt, A. Bateman, R. Evans, A. Pritzel, M. Figurnov, O. Ronneberger, R. Bates, S.A.A. Kohl, A. Potapenko, A.J. Ballard, B. Romera-Paredes, S. Nikolov, R. Jain, E. Clancy, D. Reiman, S. Petersen, A.W. Senior, K. Kavukcuoglu, E. Birney, P. Kohli, J. Jumper, and D. Hassabis, Highly accurate protein structure prediction for the human proteome. Nature 596 (2021) 590-596.

[13] A. Waterhouse, M. Bertoni, S. Bienert, G. Studer, G. Tauriello, R. Gumienny, F.T. Heer, T.A.P. de Beer, C. Rempfer, L. Bordoli, R. Lepore, and T. Schwede, SWISS-MODEL: homology modelling of protein structures and complexes. Nucleic Acids Res 46 (2018) W296-W303.

[14] E. Jurrus, D. Engel, K. Star, K. Monson, J. Brandi, L.E. Felberg, D.H. Brookes, L. Wilson, J. Chen, K. Liles, M. Chun, P. Li, D.W. Gohara, T. Dolinsky, R. Konecny, D.R. Koes, J.E. Nielsen, T. Head-Gordon, W. Geng, R. Krasny, G.-W. Wei, M.J. Holst, J.A. McCammon, and N.A. Baker, Improvements to the APBS biomolecular solvation software suite. Protein Sci 27 (2018) 112-128.
