# Supplementary material for: Case report: A novel variant (H49N) in Myelin Protein Zero gene is responsible for a patient with Charcot–Marie–Tooth disease
Source: Front Neurol. 2024 Feb 28;15:1319962. doi: 10.3389/fneur.2024.1319962 (PMC10936578; doi:10.3389/fneur.2024.1319962)
Supplement: Supplementary file 1 [file Data_Sheet_1.zip › SupplementaryTable1.DOCX]

Supplementary Table 1: 49 variants obtained through data filtering of whole exome sequencing".

| **Gene** | **Chromosome Position** | **Transcript Exon** | **Nucleotide Amino Acid** | **Homozygous/Heterozygous** | **Normal Population Frequency** | **Pathogenicity Analysis** | **Disease/Phenotype (Inheritance)** |
| --- | --- | --- | --- | --- | --- | --- | --- |
| MPZ | chr1:161277137 | NM_000530.8;exon2 | c.145C>A(p.His49Asn) | het | - | Likely pathogenic | 1. Charcot-Marie-Tooth disease type 2I (CMT2I)(AD);  2. Charcot-Marie-Tooth disease type 2J (CMT2J)(AD);  3. Dominant intermediate Charcot-Marie-Tooth disease (AD);  4. Congenital hypomyelinating neuropathy type 2 (AD);  5. Charcot-Marie-Tooth disease type 1B (CMT1B)(AD);  6. Roussy-Levy syndrome(AD);  7. Dejerine-Sottas disease (AD;AR) |
| USP45 | chr6:99894012 | NM_001080481.3;exon14 | c.1636A>T (p.Lys546Ter) | het | 0.003538 | Uncertain | ? Liberfarb syndrome congenital cataracts 19 (AR) |
| USP45 | chr6:99893693 | NM_001080481.3;exon14 | c.1955A>G (p.Gln652Arg) | het | 0.0045927 | Uncertain | ? Liberfarb syndrome congenital cataracts 19 (AR) |
| USP45 | chr6:99893698 | NM_001080481.3;exon14 | c.1950G>T (p.Lys650Asn) | het | 0.0045927 | Uncertain | ? Liberfarb syndrome congenital cataracts 19 (AR) |
| VDR | chr12:48238390 | NM_001364085.2;exon10 | c.1423C>T (p.Gln475Ter) | het | - | Likely pathogenic | Vitamin D-resistant rickets type 2A (AR) |
| AARS2 | chr6:44279256 | NM_020745.4;exon3 | c.452T>C (p.Met151Thr) | het | 0.0000544 | Likely pathogenic | 1. Combined oxidative phosphorylation deficiency 8 (AR);  2. Leukoencephalopathy with ovarian failure (AR) |
| CD36 | chr7:80302116 | NM_001001547.3;exon12 | c.1156C>T (p.Arg386Trp) | het | 0.0051348 | Likely pathogenic | 1. Coronary heart disease susceptibility type 7 (-);  2. Platelet glycoprotein IV deficiency (AR);  3. G6PD deficiency-induced resistance to malaria (-) |
| CPS1 | chr2:211457666 | NM_001875.5;exon11 | c.1150C>G (p.Pro384Ala) | het | - | Uncertain | Citrullinemia type I (AR) Neonatal pulmonary hypertension susceptibility type (-) |
| TBCE | chr1:235564898 | NM_003193.5;exon3 | c.181T>A (p.Cys61Ser) | het | 0.0002 | Uncertain | 1. Kenny-Caffey syndrome type 1 (AR);  2. Hypoparathyroidism retardation and dysmorphism syndrome (AR);  3. Progressive encephalopathy with muscle atrophy and optic atrophy (AR) |
| D2HGDH | chr2:242695408 | NM_152783.5;exon9 | c.1285G>A (p.Val429Met) | het | 0.0001096 | Uncertain | D-2-hydroxyglutaric aciduria (AR) |
| DYNC2H1 | chr11:103047038 | NM_001080463.2;exon37 | c.5749C>T (p.Arg1917Trp) | het | 0.0017 | Uncertain | Short-rib thoracic dysplasia with or without polydactyly type 3 (AR) |
| AGRN | chr1:983219 | NM_198576.4;exon22 | c.3695G>A (p.Arg1232His) | het | 0.0000408 | Uncertain | Congenital myasthenic syndrome type 8 (AR) |

AD: Autosomal Dominant; AR: Autosomal Recessive.
