# Supplementary figures and images for: Case report: A novel variant (H49N) in Myelin Protein Zero gene is responsible for a patient with Charcot–Marie–Tooth disease
Source: Front Neurol. 2024 Feb 28;15:1319962. doi: 10.3389/fneur.2024.1319962 (PMC10936578; doi:10.3389/fneur.2024.1319962)

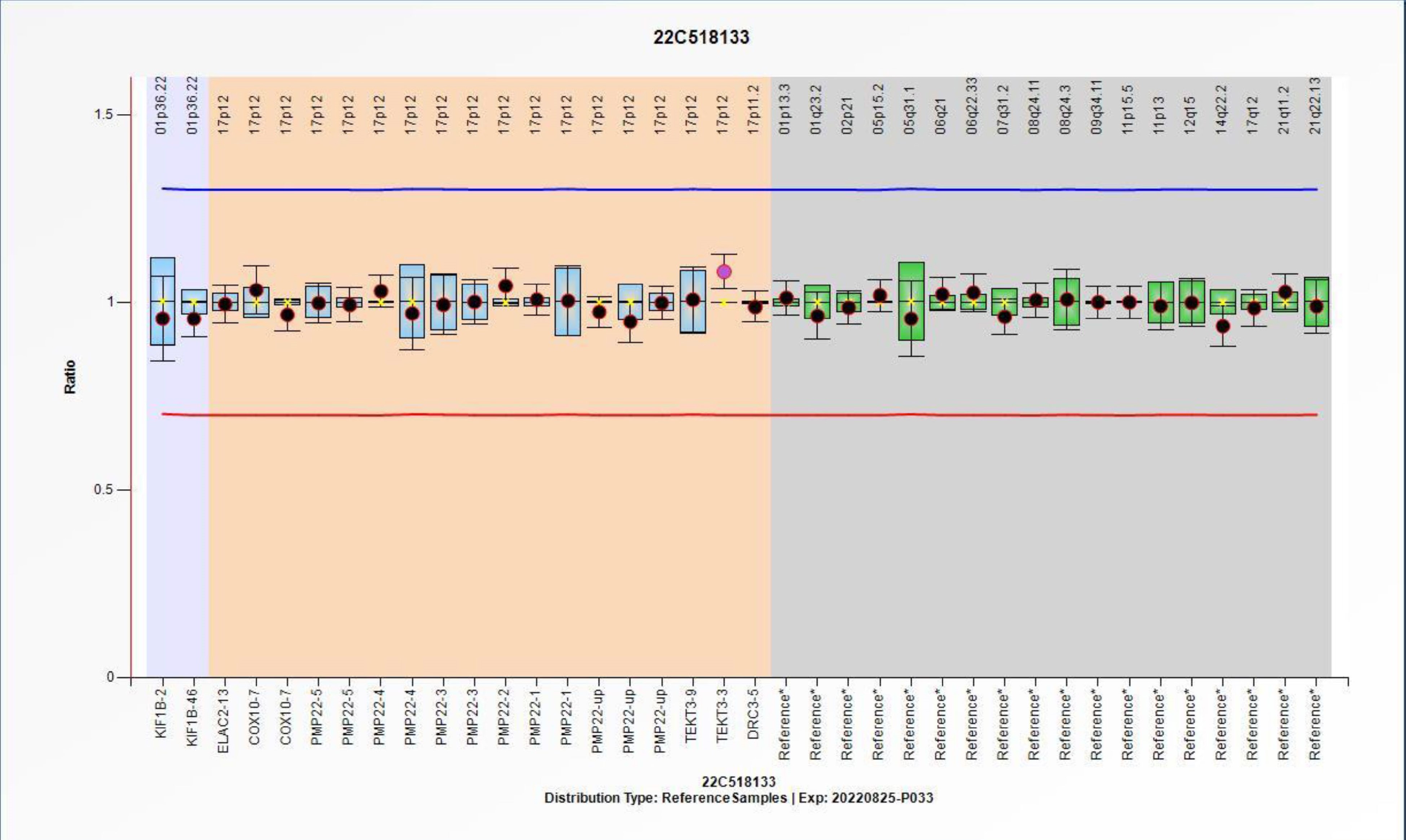

Supplement: Supplementary file 1 [file Data_Sheet_1.zip › FigureS1.JPEG]
